# Low-income minority patient engagement with automated telephonic depression assessment and impact on health outcomes

**DOI:** 10.1007/s11136-014-0900-8

**Published:** 2014-12-28

**Authors:** Irene Vidyanti, Brian Wu, Shinyi Wu

**Affiliations:** 1 Office of Health Assessment and Epidemiology, Los Angeles County Department of Public Health, Los Angeles, CA USA; 2 Keck School of Medicine, University of Southern California, Los Angeles, CA USA; 3 School of Social Work and Epstein Department of Industrial and Systems Engineering, University of Southern California, Los Angeles, CA USA; 4 RAND, Santa Monica, CA USA; 5 Schaeffer Center for Health Policy and Economics, University of Southern California, Los Angeles, CA USA

**Keywords:** Patient engagement, Diabetes complications, Depression, Clinical trial, Prevention and control

## Abstract

**Purpose:**

We investigated dimensions of low-income minority patient engagement in the context of diabetes-depression care-management with automated telephone assessment (ATA) calls as a facilitator.

**Methods:**

Mixed method analyses (including regression analyses and coding of interviews) were used to examine patient engagement with technology, assess its impact on health outcomes and satisfaction with care, and analyze barriers to engagement. Patient engagement was measured by analyzing patient’s ATA call response rates. We then evaluated those results in the context of the outcomes of the broader clinical trial, which compared three study arms.

**Results:**

Average completed call rate throughout the study is about 50 % and decreases after 6 months. The biggest barrier to patient engagement is timing. Patient engagement levels differ by baseline depression status and have no significant effect on health outcomes and satisfaction with care at 6, 12, and 18 months. However, from the preliminary clinical trial results, the arm in which the ATA system is implemented has higher satisfaction with depression care than the two control arms. Thus, it is more likely that technology positively affects satisfaction with depression care outcomes through provider engagement rather than patient engagement.

**Conclusions:**

The study’s patient engagement results and identified barriers would be useful to aid the design and implementation of future automated screening and monitoring systems to optimize patient engagement. The results also suggest that implementing a technology-supported care-management might not result in outcome disparities among patients with different levels of patient engagement.

**Electronic supplementary material:**

The online version of this article (doi:10.1007/s11136-014-0900-8) contains supplementary material, which is available to authorized users.

## Introduction

Diabetes doubles the risk of comorbid depression, especially among low-income minority patients [1–3]. The high prevalence of depression with concurrent diabetes increases patient disability and need for social support, while negatively impacting treatment efficacy, medication adherence, risk of hospitalization, self-care-management, patient–physician communication, and quality of life [4–6].

Patients with a more active involvement in their health care tend to have better outcomes and lower costs [7, 8]. Telehealth and Health Information Technology (HIT) hold promise for increased patient involvement with care through treatment monitoring and self-care behavior prompting [9]; however, the role of patient engagement with technology needed to be studied to elucidate better design and implementation of telehealth to optimize patient engagement, utilization, and health outcomes. For low-income minority patients, one concern is whether implementing telehealth will reduce or increase outcome disparities among this disadvantaged population.

Patient engagement has been defined as a broad concept including “activation; the interventions designed to increase activation; and patients’ resulting behavior,” where activation “emphasizes patient’s willingness and ability to take independent actions to manage their health and care” [7]. In the telehealth domain, patient engagement has been measured in terms of patients’ utilization and interaction with the technology through which the telecare is conveyed, i.e., in terms of usage and usage patterns [10–12].

In this paper, we studied patients’ willingness to manage their mental health within the telehealth domain by investigating patients’ usage and usage patterns of a depression telescreening and telemonitoring system. Specifically, we investigated dimensions of patient engagement in the context of safety-net diabetes-depression care-management with an automated telephone assessment (ATA) as a facilitator. Patient engagement was measured by analyzing each patient’s ATA call response rates (percentage of completed or incomplete calls divided by percentage of automated call attempts), and their usage patterns over time (call response rates over time) were also investigated. We used the patient population from the technology arm of the Diabetes-Depression Care-Management Adoption Trial (DCAT) [13], which compared three different delivery modes of depression care, including a technology arm that utilized the ATA system.

The ATA system provides an innovative way to increase patient engagement to provide critical data for cost-efficient and effective clinical care to them [13, 14]. To ensure the success of such systems in the future, we need to understand the level of patient engagement in such systems, impact on health outcomes, and barriers to patient engagement.

Such knowledge will improve the design and implementation of future systems, which in turn will enable timely access to patient-reported health conditions and thus more timely interventions to improve health outcomes.

## Methods

### Study overview and design

The DCAT clinical trial used a comparative effectiveness research design to conduct a quasi-experimental study comparing three delivery models for depression care in three groups: usual care (UC), supported care (SC), and technology-facilitated care (TC). The UC group represented the status quo of safety-net clinical practice. The SC and TC groups provided care through the Los Angeles County Department of Health Services (DHS) disease management program (DMP), which uses team staff (such as nurse practitioners, nurses, and social workers) to support chronic care-management. The clinical trial has been described in a previous paper [13].

**Fig. 1:**
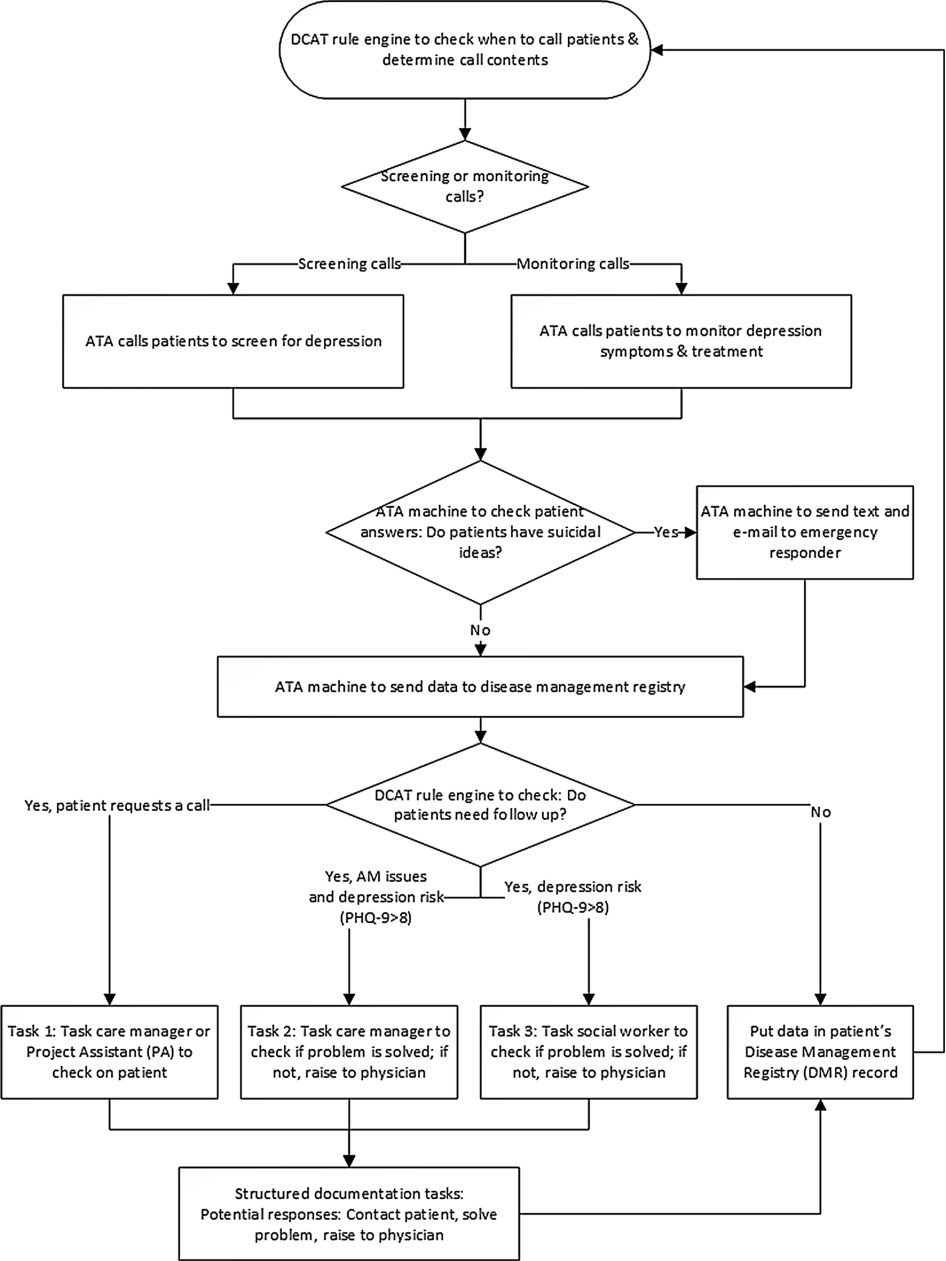
DCAT technology-facilitated care-management workflow and escalation system. (ATA = Automated telephone assessment)

The TC arm tested a patient-centered ATA as a new approach for depression care-management implementation. The system uses advanced, scalable technology to collect periodic patient-reported health data with diabetes patients at risk for depression. These results are automatically integrated with a disease registry and are used to prompt the providers to facilitate more timely care-management to patients in need. Figure 1 illustrates the DCAT technology-facilitated care-management workflow and escalation system in the TC arm. The ATA system properties have been evaluated [14].

In the trial, the ATA calls consisted of six independent modules of assessment, including depression symptom monitoring using Patient Health Questionnaire 9-item scale (PHQ-9), pain monitoring, self-care behavior prompting, medication adherence assessment, behavioral therapy practice monitoring, and patient request for provider to contact them. This modulated design enabled a customized call to each patient, as each module could be automatically activated based on patients’ current information from the patient registry. Details on the activation criteria for each module have been described in a previous paper [14].

The ATA system was used to screen and monitor depression symptoms of type 2 diabetes patients receiving DMP care in three safety-net ambulatory care clinics contained within DHS. These clinics made up the TC arm, which included 444 patients enrolled in the study. Patients received ATA calls in their language of preference: English or Spanish. Patients were in the study for 18 months. In the first 6 months, patients were in the DMP supported with the ATA technology in which the DMP providers were notified and tasked in near real time to address patients’ care needs. After 6 months, patients graduated from the DMP and returned to their usual primary care. In months 7 through 12, they still received calls from the ATA system, but only selected results (e.g., symptoms of major depression) from the ATA calls were emailed to patient’s primary care providers. Finally, in month 13 through 18, the patients remained in usual primary care and no longer received ATA calls. We conducted patient interviews to evaluate health outcomes and satisfaction with care at baseline and at the end of each of these time periods: at 6, 12, and 18 months after enrollment in the study.

#### Patient recruitment

Patients for the DCAT study were identified from database and clinic records and recruited from eight DHS primary care clinics. The TC arm patients were recruited from three of the eight primary care clinics. The patients for the study were predominantly low-income, low-literacy, middle-aged, Spanish-speaking Hispanic or Latino women who had been diagnosed with diabetes for more than 5 years. Patients were eligible for the study if they were aged 18 years or older with type 2 diabetes, had a working telephone number, spoke English or Spanish, and read and understood the consent form. Table 1 outlines the demographics and diabetes and depression characteristics of the enrolled patients in the TC arm. The study’s human subject protection protocols were reviewed and approved by the Health Science Campus IRB of the University of Southern California, and by Biomedical IRB and Education and Research Institute IRB of the University of California, Los Angeles.

**Table 1:** Baseline patient demographics and characteristics (*N* = 444)

	Numbers (%) for categorical variables; mean (SD) for continuous variables
Female	273 (62 %)
Age	52.59 (8.90)
Hispanic/Latino	400 (91 %)
A1C value	9.69 (1.94)
On insulin treatment	288 (65 %)
Toolbert diabetes self-care in the past 7 days (range 0–7)	4.23 (1.24)
PHQ-9 score 10 or greater	116 (26 %)
PHQ-9 total score (possible range 0–27)	6.37 (5.95)

### Outcome assessment

Mixed method analyses were used to examine patient engagement with technology, assess its impact on health outcomes and satisfaction with care, and analyze barriers to engagement. All outcome assessments focused on patients in the TC arm, which was the intervention arm in the DCAT trial that utilized the ATA system. However, we also used a systems perspective to analyze the patient engagement outcome assessment results by interpreting them within the context of preliminary outcome results for the whole trial.

#### Assessing effect of call characteristics and ATA program duration on patient engagement

Patient engagement was measured by analyzing each patient’s ATA call response rates (percentage of completed or incomplete calls divided by percentage of automated call attempts). Completed calls were defined as calls in which patients were reached and answers to all questions were recorded. Incomplete calls with and without Patient Health Questionnaire (PHQ)-9 indicated differing levels of patient engagement: Incomplete with PHQ-9 indicates that patients picked up the call and answered all PHQ-9 questions but did not complete the rest of the call (i.e., did not finish answering the rest of the modules in the call), while incomplete without PHQ-9 indicates that patients picked up the call and did not answer all PHQ-9 questions before hanging up.

To evaluate how call characteristics and duration on the ATA program affect patient engagement, we assessed whether patient engagement levels differed between English and Spanish language calls, whether patient engagement levels differed between calls with different numbers of modules, and whether patient engagement levels changed over time.

As aforementioned, patients received ATA calls in the first 12 months they were in the study, but after 6 months, the ATA call results were no longer integrated with their DMP care as patients graduated from the DMP program after 6 months. Therefore, we wanted to examine whether patient engagement levels in the technology could be sustained beyond their participation in intensive DMP care-management, but with limited involvement of primary care physicians. To do that for each patient, we tabulated the number of completed calls and total calls received in the first 6 months and second 6 months they participated in the study, and calculated the percentage of completed calls for each period. Then, we averaged the percentage of completed calls across all patients for each period to obtain and compare the percentage of completed calls in the first 6 months and the second 6 months of the study.

#### Barriers to patient engagement

To analyze barriers to patient engagement, patients with incomplete calls were followed up with a human call to determine reasons for failure to contact. We then coded and tabulated the reasons for incomplete calls from these patient interviews. Additionally, to gain a more complete picture of patient engagement, we also obtained the providers’ perspectives on supports and barriers to patient engagement by interviewing providers in the study clinics. We conducted semi-structured, open-ended interviews with the providers and analyzed the responses to find common themes on possible systemic barriers to patient engagement.

#### Statistical analyses to evaluate effects of patient engagement

Statistical analyses were performed to evaluate whether patient engagement levels differed by baseline depression status, and to assess the effect of patient engagement levels on health outcomes and satisfaction with care at 6, 12, and 18 months, controlled for baseline measures. To do this, the completed call rate for each patient was calculated, and a binary indicator for having *a* > 50 % completed call rate created as a variable indicating patient engagement.

To evaluate whether patient engagement levels differed by baseline depression status, logistic regression was performed, with the dependent variable being *a* > 50 % completed call rate and the independent variable being baseline depression status (depressed = PHQ-9 at baseline >10), controlling for baseline covariates (age, preferred language, gender, insulin, onset age of diabetes, BMI, study clinic).

To assess the effect of patient engagement levels on health outcomes and satisfaction with care at 6, 12, and 18 months, we performed logistic (for binary outcomes) or ordinary linear regression (for continuous outcomes), with the dependent variable being the health outcome or satisfaction with care in question at 6, 12, and 18 months, and the independent variable being the >50 % completed call rate, controlled for baseline covariates and the baseline value of the health outcome and satisfaction with care being assessed.

#### Patient engagement results within the context of the results of the overall clinical trial

To obtain a fuller picture of how the patient engagement results in the preceding sections fit within the context of the larger clinical trial, we presented some preliminary outcome results from the clinical trial. The preliminary outcomes evaluation compared the depression outcomes and satisfaction with depression care between the technology-supported intervention arm and the two control arms in the clinical trial. A generalized propensity score method for interpreting multiple-intervention-arm quasi-experimental studies was applied to analyze the 6-month outcome. Baseline characteristics that might influence outcomes were used to estimate the propensity score, and then the propensity scores were used in regression models as covariates to predict comparative treatment effectiveness.

Specifically, by analyzing the patient engagement analysis results through the lens of the outcome evaluation in the larger context, we could understand the pathway through which the technology-supported care-management intervention impacted health outcomes, and could determine whether patient engagement is an important part of that pathway.

## Results

### How call characteristics and duration on the ATA program affect patient engagement

#### Rates of patient engagement

During the study, 3,180 automated calls were made during an 18-month period (10/20/2011–7/4/2013). Of these, around 50 % were completed calls, 20 % were incomplete (either with or without PHQ-9), and 30 % (coded as “others” in Table 1) were not picked up or reached (disconnected numbers, expired call attempts, or reached answering machine) or were rescheduled (Table 1). The majority of incomplete calls (84 %) were incomplete without PHQ-9.

The rates of completed calls were similar for English calls and Spanish calls. The rate for total incomplete calls was slightly higher for Spanish calls than for English calls.

#### Rates of patient engagement with number of modules asked

As can be seen in Table 2, the rates of completed calls and incomplete with PHQ-9 calls were highest for calls in which six modules are asked. Patients who were not on anti-depressive medication (AM) or psychotherapy were asked four modules, and those on AM or psychotherapy were asked five modules. Those on AM and psychotherapy were asked all six modules. Patients asked all six modules may have been more motivated (thus their engagement in two forms of depressive treatment) and consequently may have had a higher degree of patient engagement.

**Table 2:** Rates of patient engagement by language and number of modules asked

Call status	Language			No. of modules		
	English	Spanish	Grand total	4	5	6
Complete	48.79 %	49.04 %	48.99 %	49.37 %	47.00 %	59.15 %
Incomplete w/o PHQ-9	24.14 %	26.54 %	26.10 %	26.50 %	25.25 %	22.54 %
Incomplete w/PHQ-9	3.45 %	3.58 %	3.55 %	3.77 %	3.00 %	2.82 %
Others	23.62 %	20.85 %	21.35 %	20.36 %	24.75 %	15.49 %
Total calls	580	2,600	3,180	2,309	800	71

#### Rates of patient engagement over time

The rate of completed calls was higher for patients who were in the first 6 months of the study than for patients in the second 6 months of the study (Table 3). Patients’ motivation to complete calls might have decreased over time due to boredom from the repetition of the calls or less interaction with their providers (since patients leave the DMP after 6 months in the study and DMP providers no longer follow-up with patients based on the results of the ATA calls). This potentially indicates the importance of having provider support the ATA technology to keep patient engaged.

**Table 3:** Rates of patient engagement over time

Time in the study	Number of complete calls	Number of calls	(%) complete calls
Month 0–6	875	1,582	55.31
Month 7–12	683	1,598	42.74

### Patients’ perspective: barriers to patient engagement

All patients with incomplete calls were contacted for this analysis, and 282 out of 284 patients (99 %) were reached. Of these 282 patients, 43 % cited inconvenient call time as the reason for not completing the automated call, whereas 57 % of patients did not complete the automated calls due to preference for human calls, bad cell phone connection, disconnected phone, non-working phone, and/or personal reasons. In other words, most incomplete calls were due to timing issues for the call (patients were busy at the time of the call or were not at home, or other timing issues). Although there is a larger segment of issues due to the need for human interaction and technology issues, the greatest single reason for not reaching patients is timing issues. Reasons for failure to complete the calls and the incidence for each reason are tabulated below.

### Providers’ perspective on patient engagement

A total of 12 interviews were conducted with providers in the technology-supported care arm: three social workers, a medical doctor, two registered nurses, a nurse practitioner, and two physicians’ assistants, in addition to three site clinic/leadership staff.

Three themes related to patient engagement emerged in the interviews: perceived supports for patient engagement, perceived barriers for patient engagement, and the shift of care behavior toward increased engagement with patients and toward increasing patient engagement.

In terms of perceived supports for patient engagement, providers conveyed that patients felt like someone was “on their side” and that they had care providers looking out for them beyond usual care. According to providers, patients felt an improved sense of connection especially because they felt that they could always reach someone when needed.

An excerpt from the interview on this theme is given below:

A: I think the DCAT program is good in evaluating depression in all patients with chronic disease and also in giving them the sense that there is someone beyond their primary care physician who can facilitate care for them, who they could reach out to in times of stress or depression or whatever, and get the attention that they needed in a timely fashion.

In terms of perceived barriers for patient engagement, providers stated that patients sometimes were unaware of why these calls were coming because of a possible lack of clear instructions during recruitment or that they simply forgot. This is supported by the results from our follow-up with patients with incomplete calls, in which 20 patients cite study-related reasons (lack of clear instructions, forgot about being in a study, and so on) as the reasons for incomplete call.

An excerpt from the interview on this theme is given below:

Q: And what would you change? I mean we talked about several things.

A: Yeah just the phone thing, the disclosure to the patients. I really you know and again I wasn’t in the room when the recruiters talked to them but I think some patients will say yes to anything. They will. I mean I call them by the wrong name and they say yes and then I find out it’s the wrong name. I think it has to be like almost making sure that they’re looking in your eyes saying, ‘Do you understand this is going to be in your house? They’re going to call’. And then later when they call, I would say, you knew they were going to call. They tell you, ‘Oh we didn’t understand or I didn’t know what she meant.’ You know, so I don’t know if it’s a signature they need or just the disclosure. Once they know, I think they’re okay that this is what’s going to happen.

Finally, providers also reported that the technology helped shift their care behavior from focusing on screening and monitoring to treating patients who need it, increasing their engagement with patients, as can be seen in the excerpt below:

A: I really think on our side, I think it was great for the patients. We really were more proactive with medication. We were more zoned in on what does, you know, how is this going to affect the patient? Where before it was like, oh they scored a two, okay let’s go onto the next level.

This shift of some of the screening and monitoring burden to technology also allows some providers to spend more time getting patients to be more engaged in their care and open up more in their interactions with the providers, as revealed in the excerpt below:

Q1: Doing this monitoring of depression, has that changed your awareness of the population?

A: Personally, for me, I think I have put in a little bit more effort to get on a little more involved in the patient so that they can express to me and feel confident that this information is just here, it’s not spoken publicly, and I’ve dealt with the issue.

Q2: So it’s kind of like building some trust so that they can be able to talk to you a little bit more.

A: Correct. And I think, after your DCAT program, I’ve been a little bit more sensitive to that aspect of it, and I really thank you for that.

### Evaluating the effect of patient engagement levels

Tables 4 and 5 show the significant results from our evaluation of the effect of patient engagement on health outcomes and satisfaction with care. Tables for outcomes that are not significant are included in the supplemental materials.

**Table 4:** Barriers of patient engagement from the patients’ perspective

Human call	Count	Percent
Wants human calls/does not like automated calls	14	4.9
*Non-study*-*related reasons*		
Busy/was not home/timing issues	122	43.1
Wrong number	10	3.5
Bad connection/got disconnected	11	3.9
Hung up by accident	2	0.7
Gave friend’s/family’s number	3	1.1
Someone else answered/does not remember receiving call	17	6.0
Phone was not working/battery dead	3	1.1
Phone does not accept incoming calls	1	0.4
Low on cell phone minutes	1	0.4
Said she did answer call (although no record of answers in DMR)/repeat entries with final being answered/states answered but on incomplete sheet	14	4.9
Called on christmas	1	0.4
Gave new number	1	0.4
*Study*-*related reasons*		
Does not want to be bothered	1	0.4
Did not remember about study/does not know what the ATA was	6	2.1
Confused about pin	1	0.4
Tech problem	4	1.4
Does not like questions	2	0.7
Graduated/dismissed/no longer goes to study clinic	3	1.1
Call confusing b/c did not say what it is for	1	0.4
Thought another study whose machine was broken	1	0.4
Thinks that after 6-month follow-up, no need to answer ATA calls	1	0.4
MRN coded twice for same person	1	0.4
*Personal reasons*		
Personal reasons (e.g., family member in hospital)	3	1.1
In pain/too sick to move/sick	9	3.2
Depressed	1	0.4
Hard of hearing	3	1.1
Not technologically savvy	2	0.7
Thought it was fraud	1	0.4
Do not want to answer over phone	1	0.4
Felt fine, not depressed	2	0.7
Disappointed in care provided	1	0.4
*Failed to locate patient*		
Failed to locate patient/incomplete	23	8.1
Disconnected	14	4.9
No entry on sheet	1	0.4
Passed away	1	0.4
Partial entry on sheet	3	1.1
Answering machine for different person	1	0.4
Total	283

**Table 5:** Odds ratio estimates for logistic regression with patient engagement (more than 50 % call completion rate) as dependent variable and depression status at baseline as independent variable, controlled for baseline covariates

Odds ratio estimates			
Effect	Point estimate	95 % Wald confidence limits	
Age	0.992	0.960	1.025
Spanish	0.797	0.474	1.340
Sex	1.063	0.702	1.610
Insulin	1.089	0.704	1.686
Onset age	0.997	0.967	1.027
BMI	0.979	0.952	1.007
Study site	1.011	0.668	1.530
Depressed at baseline	0.608	0.389	0.950

#### Evaluating whether patient engagement differs by baseline depression status

As can be seen in Table 4, patients who had a PHQ-9 score of ten or higher at baseline were less likely to be engaged with the ATA technology; i.e., depressed patients were less likely to reach >50 % completed call rate than non-depressed patients [OR = 0.608 (0.389, 0.950)].

#### Evaluating the effect of patient engagement on health outcomes and satisfaction with care

Patient engagement (having >50 % completed call rate) did not have a significant effect on depression status at 6, 12, and 18 months (*p* > 0.05 for all three time periods). Patient engagement also did not have a significant effect on patients’ diabetes self-care at 6, 12, and 18 months (*p* > 0.05 for all three time periods). Patient engagement also did not have a significant effect on patients’ satisfaction with depression care and satisfaction with diabetes care at 6, 12, and 18 months (*p* > 0.05 for all three time periods).

### Putting the patient engagement results in the context of the results of the overall clinical trial

Our analyses of the overall clinical trial (not shown) found both TC and SC significantly improved satisfaction with care for emotional problems (at *p* < 0.05). Moreover, the difference between TC and SC is also significant (at *p* < 0.05), indicating that patients in the TC arm were significantly more satisfied with their depression care than those in either SC or UC.

Comparing the three arms, TC resulted in better satisfaction with depression care outcomes at 6 months than the control groups (SC and UC). However, patient engagement within the TC group did not affect this outcome significantly within the TC group. Our other analysis of patient satisfaction with the ATA calls can shed some lights on this result (manuscript in preparation). At the 6- and 12-months follow-up interviews, the vast majority of the TC patients (80 % or more) perceived the ATAs as being “usually” or “always” easy to use. Three quarters of the patients did not feel that the ATA calls were a bother. However, the percentages of patients who felt that the ATAs were “usually” or “always” useful in empowering them to access providers or to remind them of self-care activities were moderate: average at 61 % at 6 months and 49 % at 12 months.

Thus, it is likely that the DCAT technology does not positively affect satisfaction with depression care outcomes through patient engagement but through other pathways, since patient engagement does not affect satisfaction with depression care significantly in the TC group, and preliminary results on the patient satisfaction with the ATA calls indicate that patients’ perception of the direct usefulness of ATA system to them is only moderate. Instead, it is possible that the DCAT technology affects satisfaction with depression care positively by increasing provider engagement through the integration of the ATA technology results into the care-management system. For example, tasks automatically generated based on ATA call results might have resulted in more provider-involved care for all patients in TC group regardless of each patient’s engagement with the technology and may have led to higher satisfaction with depression care for all patients in the TC group without a significant differential effect due to differing levels of patient engagement.

In other words, based on these results, we hypothesize that rather than increasing patient engagement directly, the pathway by which TC improves satisfaction with depression care results is an increase in provider engagement, perhaps through the change in their clinical microsystems of how they identify and treat patient care needs due to technology support in care-management. This increase in provider engagement then possibly increases patient engagement.

Some insights from the provider interview lend support to this hypothesis. As shown in some of the excerpts in section “Evaluating the effect of patient engagement levels,” some providers feel that the DCAT technology makes them more sensitive to patient needs and having the technology support the screening and monitoring allows them to spend more time adjusting depression therapy, increasing their engagement. Also, it makes them more sensitive to patient needs and gets them to try to increase patient interaction during care appointments, increasing patient engagement.

In addition, preliminary results from surveys of the providers in the study indicate that providers in the technology arm have higher outcome expectancy and self-efficacy than those in the non-technology arm [15]. Both of these factors are linked to increased engagement and better performance in social cognitive theory [16, 17]. In terms of the manifestation of these factors in provider behavior, the results of the survey back the insights from the provider interviews: more providers in the technology arm educate patients about depression, discuss management options, monitor adherence and side effects, and adjust depression therapy compared to those in the non-technology arms, while they assess for depressive episodes less (likely due to the shift of the burden of depression assessment to the ATA calls in the technology arm) [15]. These shifts in providers’ outcome expectancy and self-efficacy, as well as the shift in provider behavior from assessment to education and care-management (which may facilitate shared decision making) in the technology arm might be the reason why patients in turn are more satisfied with care in the TC arm. This hypothesis would need to be empirically evaluated in future research.

Compared to UC, both TC and SC are shown to significantly improve depression outcomes, and TC is shown to significantly improve satisfaction with diabetes care. However, TC is not significantly different to SC in terms of depression outcomes and satisfaction with diabetes care. Nevertheless, it is important to note that this does not mean that the DCAT technology might not have any impact on these particular outcomes compared to SC, because DCAT technology integrated care in a way that changed the providers’ workflow; therefore, as the DMP care delivery in TC was different from care delivery in SC. Providers in the TC arm were able to shift their care to those who needed it more because screening and monitoring for low-risk patients were supported by the technology [15]. DCAT technology to support DMP care might be a viable option to the usual care in overstretched DMP clinics.

That the patient engagement results do not show significant effect of patient engagement on these outcomes in the TC group indicates that the implementation of such a technology for depression care to support DMP care might not result in disparity of outcomes among patients of differing levels of patient engagement. However, there might be a minimum threshold of overall patient engagement in the system for the results to hold; i.e., there might be a need for enough patients to be engaged to affect provider engagement significantly such that providers’ shift in workflow due to the DCAT technology will not impact outcomes negatively. What this minimum threshold might be warrants further study.

## Discussion

A question that surfaces from the results is whether there is a minimum threshold required for patient engagement in ATA-supported systems for the system to benefit as a whole. This requires further study. However, if the minimum threshold indeed exists and patient engagement levels do not have a significant effect on health outcomes at the individual level, then the implication is that we may not need to activate patients to be engaged at the individual level since there is no disparity of care that results from differing levels of patient engagement once the minimum threshold is met. Rather, less expensive and simpler-to-implement activation efforts that target the entire group of patients may be appropriate.

Another metric of patient engagement that we considered with this dataset is the number of requests to talk to nurse/social worker made through the ATA calls, as this might be an indication of patients being more active in gaining information for their depression care. However, using that metric is problematic since we find that many of these requests were accidental or they actually meant to talk to the study assistant instead. Also, anecdotal evidence from providers in the study clinic also suggest that many patients either call the clinic or call the direct line or mobile number of particular health care providers directly instead of going through the ATA to request to talk to a nurse/social worker, making the considered metric not reflective of the actual number of interactions patients have with their providers, since we do not have a record of the direct calls made outside the ATA system.

We are also analyzing the results of patient surveys in the TC arm that examines the impact of ATA on patient activation, i.e., how the ATA helps in making them feel more active and empowered in their depression care (by being more aware of how one is feeling, being reminded to take care of health, feeling better connected with providers). The preliminary results, as aforementioned, show moderate effects, albeit further analysis is still ongoing. This work, which examines another aspect of patient engagement piece, will complement the results of this paper in painting a better picture of the role of patient engagement in the DCAT study.

Diabetes and depression, as chronic diseases, require preventive and self-care activities, and the way care is delivered and patient is engaged can influence patients’ self-care abilities [18]. Thus, even though our study did not demonstrate patient engagement to be an important predictor in the TC group, it is still important to examine barriers to engagement, as the results would also apply to other automated call systems to support care. Additionally, learning about these barriers will help us understand how to change the ATA system design and delivery to remove some of these barriers; this will be important if there is indeed a minimum threshold required for patient engagement, and a new system needs to ensure that the threshold is met.

As an example, most patients with incomplete calls cited timing issues as a major barrier, even though the ATA calls were adapted to specific patient time preferences, and multiple calls were made if patients were not reached with the first call. Patients were asked their preferred call day and time when ATA calls were configured. However, when the first call was missed, the ATA system tried again once every 3 h between 8 AM and 8 PM regardless of call time preference, and these callback times might not have been convenient to patients. Possible solutions to reduce the barrier to patient engagement due to timing issues may include allowing patients to call back at a time of their own choosing if calls are missed, or configuring the system to call back only at times that are convenient for each individual patient.

About 10 % of patients with incomplete calls cited study-related reasons as the barrier. To reduce this barrier, patients should be given instructions that are easy to read, understand, and remember. These instructions could be given either during the recruiting session for the study or during enrollment into the program. This solution would also be relevant if such a system was to be implemented in the real-world setting, as it highlights the need to provide clear instructions and provide reminders to answer the calls to patients in the beginning of the implementation.

The system should also take into account personal reasons for incomplete calls and possibly personalize the ATA system further to accommodate different needs of patients. For instance, hard-of-hearing patients could potentially be provided specific prompts, and patients who are sick could be given calls or monitoring to receive calls at a more optimal time.

At the second level of engagement in the framework for patient and family engagement in health care, organizations should “reach out for consumer input to ensure that they will be as responsive as possible to patients’ needs.” [19]. Thus, future iterations of ATA should incorporate refinements from the patient feedback obtained through post-study evaluations such as this study, but also through pre-study patient input such as focus groups to determine how to best design the technology to meet patient needs.

It is also worth noting that levels of patient engagement decline after the first 6 months. It seems that to sustain patient engagement over the long run, provider support, or other ways to activate patient engagement may be necessary.

The results of our patient engagement analysis, put in the context of the preliminary outcome results from the DCAT trial, highlight the possibility that provider engagement plays an important role in the pathway of the impact of the DCAT system on outcomes. Our current, ongoing work involves analyzing provider engagement in the DCAT study to investigate this pathway. This pathway might explain why the system as a whole may benefit (in terms of satisfaction with care in this case) despite disparities in levels of patient engagement within the system, as the increased provider engagement benefits all patients in the clinics.

## Conclusions

In this paper, we have investigated various dimensions of patient engagement with an ATA technology, examined barriers to patient engagement from the patients’ and providers’ perspective, assessed the impact of patient engagement levels on health outcomes and satisfaction with care, and evaluated the results in the broader context of the clinical trial results. The patient engagement results and barriers that we found would aid the design and implementation of future automated screening and monitoring systems to optimize patient engagement.

Our results also raise an interesting implication that implementing a technology-supported care-management might not result in disparity of outcomes among patients of different levels of patient engagement and that the system may benefit as a whole despite disparities in levels of patient engagement within the system. Our analysis also hints at a pathway in which satisfaction with depression care is improved through increased provider engagement, which consequently might increase patient engagement; this hypothesis should be empirically evaluated in future research.

## Electronic supplementary material

Below is the link to the electronic supplementary material.

Supplementary material 1 (DOCX 22 kb)
