# Supplementary material for: Low-income minority patient engagement with automated telephonic depression assessment and impact on health outcomes
Source: Qual Life Res. 2014 Dec 28;24(5):1119–29. doi: 10.1007/s11136-014-0900-8 (PMC4412647; doi:10.1007/s11136-014-0900-8)
Supplement: Supplementary file 1 — Supplementary material 1 (DOCX 22 kb) [file 11136_2014_900_MOESM1_ESM.docx]

**Supplementary materials**

Table 1. Odds ratio estimates for logistic regression with depression status at 6 months as dependent variable and patient engagement as independent variable, controlled for baseline covariates.

| **Odds Ratio Estimates** | | | | **Pr > ChiSq** |
| --- | --- | --- | --- | --- |
| **Effect** | **Point Estimate** | **95% Wald Confidence Limits** | |  |
| **Age** | 0.987 | 0.930 | 1.047 | 0.6639 |
| **Spanish language preference** | 1.257 | 0.458 | 3.449 | 0.6563 |
| **Sex** | 0.764 | 0.356 | 1.643 | 0.4913 |
| **On insulin** | 1.127 | 0.512 | 2.480 | 0.7667 |
| **Diabetes onset age** | 1.017 | 0.963 | 1.073 | 0.5544 |
| **BMI** | 1.068 | 1.017 | 1.122 | 0.0083 |
| **Study site** | 1.130 | 0.548 | 2.327 | 0.7412 |
| **Depressed at baseline** | 10.163 | 5.009 | 20.619 | <.0001 |
| **Having >50% complete call rate** | 0.766 | 0.380 | 1.547 | 0.4577 |

Table 2. Odds ratio estimates for logistic regression with depression status at 12 months as dependent variable and patient engagement as independent variable, controlled for baseline covariates.

| **Odds Ratio Estimates** | | | | **Pr > ChiSq** |
| --- | --- | --- | --- | --- |
| **Effect** | **Point Estimate** | **95% Wald Confidence Limits** | |  |
| **Age** | 1.044 | 0.993 | 1.097 | 0.0896 |
| **Spanish language preference** | 1.261 | 0.526 | 3.021 | 0.6033 |
| **Sex** | 0.533 | 0.282 | 1.007 | 0.0525 |
| **On insulin** | 1.219 | 0.626 | 2.375 | 0.5607 |
| **Diabetes onset age** | 0.972 | 0.930 | 1.016 | 0.2088 |
| **BMI** | 1.037 | 0.992 | 1.085 | 0.1108 |
| **Study site** | 1.189 | 0.643 | 2.200 | 0.5808 |
| **Depressed at baseline** | 6.016 | 3.271 | 11.066 | <.0001 |
| **Having >50% complete call rate** | 1.728 | 0.938 | 3.183 | 0.0791 |

Table 3. Odds ratio estimates for logistic regression with depression status at 18 months as dependent variable and patient engagement as independent variable, controlled for baseline covariates.

| **Odds Ratio Estimates** | | | | **Pr > ChiSq** |
| --- | --- | --- | --- | --- |
| **Effect** | **Point Estimate** | **95% Wald Confidence Limits** | |  |
| **Age** | 1.028 | 0.974 | 1.085 | 0.3105 |
| **Spanish language preference** | 2.310 | 0.753 | 7.082 | 0.1431 |
| **Sex** | 0.458 | 0.223 | 0.942 | 0.0337 |
| **On insulin** | 1.488 | 0.693 | 3.194 | 0.3079 |
| **Diabetes onset age** | 0.987 | 0.940 | 1.036 | 0.5947 |
| **BMI** | 1.033 | 0.981 | 1.087 | 0.2169 |
| **Study site** | 0.770 | 0.379 | 1.561 | 0.4678 |
| **Depressed at baseline** | 6.590 | 3.356 | 12.942 | <.0001 |
| **Having >50% complete call rate** | 1.267 | 0.644 | 2.495 | 0.4930 |

Table 4. Linear regression results with diabetes self-care at 6 months as dependent variable, patient engagement as independent variable, controlled for baseline covariates.

| **Parameter Estimates** | | | | |
| --- | --- | --- | --- | --- |
| **Variable** | **Parameter Estimate** | **Standard Error** | **t Value** | **Pr > \|t\|** |
| **Intercept** | 2.88610 | 0.58269 | 4.95 | <.0001 |
| **Age** | 0.01315 | 0.01061 | 1.24 | 0.2160 |
| **Spanish language preference** | 0.38236 | 0.17017 | 2.25 | 0.0253 |
| **Sex** | 0.15642 | 0.13197 | 1.19 | 0.2367 |
| **On insulin** | -0.06033 | 0.13774 | -0.44 | 0.6617 |
| **Diabetes onset age** | -0.00792 | 0.00978 | -0.81 | 0.4186 |
| **BMI** | -0.00754 | 0.00889 | -0.85 | 0.3971 |
| **Study site** | 0.10988 | 0.12952 | 0.85 | 0.3968 |
| **Diabetes self-care** | 0.31870 | 0.05065 | 6.29 | <.0001 |
| **Having >50% complete call rate** | 0.13690 | 0.12186 | 1.12 | 0.2620 |

Table 5. Linear regression results with diabetes self-care at 12 months as dependent variable, patient engagement as independent variable, controlled for baseline covariates.

| **Parameter Estimates** | | | | |
| --- | --- | --- | --- | --- |
| **Variable** | **Parameter Estimate** | **Standard Error** | **t Value** | **Pr > \|t\|** |
| **Intercept** | 1.10392 | 0.69782 | 1.58 | 0.1146 |
| **Age** | 0.01834 | 0.01216 | 1.51 | 0.1323 |
| **Spanish language preference** | 0.19474 | 0.20614 | 0.94 | 0.3455 |
| **Sex** | 0.12146 | 0.15386 | 0.79 | 0.4304 |
| **On insulin** | 0.15051 | 0.15957 | 0.94 | 0.3463 |
| **Diabetes onset age** | 0.00239 | 0.01132 | 0.21 | 0.8325 |
| **BMI** | -0.00256 | 0.01097 | -0.23 | 0.8154 |
| **Study site** | -0.10524 | 0.15231 | -0.69 | 0.4901 |
| **Diabetes self-care** | 0.41150 | 0.05990 | 6.87 | <.0001 |
| **Having >50% complete call rate** | 0.18465 | 0.14413 | 1.28 | 0.2010 |

Table 6. Linear regression results with diabetes self-care at 18 months as dependent variable, patient engagement as independent variable, controlled for baseline covariates.

| **Parameter Estimates** | | | | |
| --- | --- | --- | --- | --- |
| **Variable** | **Parameter Estimate** | **Standard Error** | **t Value** | **Pr > \|t\|** |
| **Intercept** | 1.75361 | 0.69864 | 2.51 | 0.0126 |
| **Age** | 0.01240 | 0.01205 | 1.03 | 0.3044 |
| **Spanish language preference** | 0.25804 | 0.20623 | 1.25 | 0.2118 |
| **Sex** | 0.12075 | 0.15913 | 0.76 | 0.4486 |
| **On insulin** | -0.05943 | 0.16393 | -0.36 | 0.7172 |
| **Diabetes onset age** | -0.00129 | 0.01126 | -0.11 | 0.9091 |
| **BMI** | -0.00654 | 0.01109 | -0.59 | 0.5562 |
| **Study site** | -0.24234 | 0.15513 | -1.56 | 0.1193 |
| **Diabetes self-care** | 0.43710 | 0.06028 | 7.25 | <.0001 |
| **Having >50% complete call rate** | 0.17020 | 0.14866 | 1.14 | 0.2532 |

Table 7. Logistic regression results with satisfaction with depression care at 6 months as dependent variable, patient engagement as independent variable, controlled for baseline covariates.

| **Odds Ratio Estimates** | | | |
| --- | --- | --- | --- |
| **Effect** | **Point Estimate** | **95% Wald Confidence Limits** | |
| **Age** | 1.033 | 0.953 | 1.119 |
| **Spanish language preference** | 0.898 | 0.220 | 3.668 |
| **Sex** | 1.987 | 0.707 | 5.584 |
| **On insulin** | 0.398 | 0.126 | 1.260 |
| **Diabetes onset age** | 0.985 | 0.919 | 1.056 |
| **BMI** | 1.014 | 0.945 | 1.087 |
| **Study site** | 0.764 | 0.290 | 2.013 |
| **Satisfaction with depression care at baseline** | 9.661 | 1.584 | 58.928 |
| **Having >50% complete call rate** | 1.296 | 0.485 | 3.461 |

Table 8. Logistic regression results with satisfaction with depression care at 12 months as dependent variable, patient engagement as independent variable, controlled for baseline covariates.

| **Odds Ratio Estimates** | | | |
| --- | --- | --- | --- |
| **Effect** | **Point Estimate** | **95% Wald Confidence Limits** | |
| **Age** | 1.024 | 0.933 | 1.125 |
| **Spanish language preference** | 0.586 | 0.049 | 7.032 |
| **Sex** | 1.124 | 0.327 | 3.863 |
| **On insulin** | 1.469 | 0.434 | 4.970 |
| **Diabetes onset age** | 0.990 | 0.915 | 1.071 |
| **BMI** | 1.017 | 0.929 | 1.115 |
| **Study site** | 1.368 | 0.403 | 4.649 |
| **Satisfaction with depression care at baseline** | 0.727 | 0.112 | 4.711 |
| **Having >50% complete call rate** | 1.322 | 0.426 | 4.103 |

Table 9. Logistic regression results with satisfaction with depression care at 18 months as dependent variable, patient engagement as independent variable, controlled for baseline covariates.

| **Odds Ratio Estimates** | | | |
| --- | --- | --- | --- |
| **Effect** | **Point Estimate** | **95% Wald Confidence Limits** | |
| **Age** | 0.957 | 0.869 | 1.053 |
| **Spanish language preference** | 1.577 | 0.126 | 19.679 |
| **Sex** | 1.952 | 0.446 | 8.535 |
| **On insulin** | 0.626 | 0.133 | 2.942 |
| **Diabetes onset age** | 1.028 | 0.948 | 1.116 |
| **BMI** | 0.950 | 0.851 | 1.060 |
| **Study site** | 3.518 | 0.776 | 15.950 |
| **Satisfaction with depression care at baseline** | 0.836 | 0.096 | 7.262 |
| **Having >50% complete call rate** | 0.275 | 0.066 | 1.143 |

Table 10. Logistic regression results with satisfaction with diabetes care at 6 months as dependent variable, patient engagement as independent variable, controlled for baseline covariates.

| **Odds Ratio Estimates** | | | |
| --- | --- | --- | --- |
| **Effect** | **Point Estimate** | **95% Wald Confidence Limits** | |
| **Age** | 0.972 | 0.913 | 1.035 |
| **Spanish language preference** | 1.185 | 0.424 | 3.312 |
| **Sex** | 0.671 | 0.285 | 1.578 |
| **On insulin** | 0.967 | 0.395 | 2.369 |
| **Diabetes onset age** | 1.044 | 0.986 | 1.106 |
| **BMI** | 1.004 | 0.951 | 1.061 |
| **Study site** | 0.653 | 0.300 | 1.425 |
| **Satisfaction with diabetes care at baseline** | 19.157 | 2.598 | 141.254 |
| **Having >50% complete call rate** | 1.179 | 0.552 | 2.519 |

Table 11. Logistic regression results with satisfaction with diabetes care at 12 months as dependent variable, patient engagement as independent variable, controlled for baseline covariates.

| **Odds Ratio Estimates** | | | |
| --- | --- | --- | --- |
| **Effect** | **Point Estimate** | **95% Wald Confidence Limits** | |
| **Age** | 1.035 | 0.976 | 1.097 |
| **Spanish language preference** | 1.706 | 0.657 | 4.431 |
| **Sex** | 1.051 | 0.502 | 2.202 |
| **On insulin** | 0.984 | 0.446 | 2.172 |
| **Diabetes onset age** | 1.013 | 0.958 | 1.072 |
| **BMI** | 1.049 | 0.990 | 1.110 |
| **Study site** | 0.816 | 0.385 | 1.728 |
| **Satisfaction with diabetes care at baseline** | 19.825 | 1.810 | 217.173 |
| **Having >50% complete call rate** | 1.206 | 0.584 | 2.488 |

Table 12. Logistic regression results with satisfaction with diabetes care at 18 months as dependent variable, patient engagement as independent variable, controlled for baseline covariates.

| **Odds Ratio Estimates** | | | |
| --- | --- | --- | --- |
| **Effect** | **Point Estimate** | **95% Wald Confidence Limits** | |
| **Age** | 0.951 | 0.888 | 1.019 |
| **Spanish language preference** | 0.391 | 0.082 | 1.860 |
| **Sex** | 1.634 | 0.640 | 4.170 |
| **On insulin** | 1.407 | 0.536 | 3.698 |
| **Diabetes onset age** | 1.049 | 0.984 | 1.118 |
| **BMI** | 0.985 | 0.922 | 1.053 |
| **Study site** | 1.523 | 0.561 | 4.137 |
| **Satisfaction with diabetes care at baseline** | <0.001 | <0.001 | >999.999 |
| **Having >50% complete call rate** | 0.991 | 0.409 | 2.403 |
